# Supplementary material for: Molecular mechanism underlying regulation of Arabidopsis CLCa transporter by nucleotides and phospholipids
Source: Nat Commun. 2023 Aug 12;14:4879. doi: 10.1038/s41467-023-40624-z (PMC10423218; doi:10.1038/s41467-023-40624-z)
Supplement: Supplementary file 1 — Supplementary Information [file 41467_2023_40624_MOESM1_ESM.pdf]

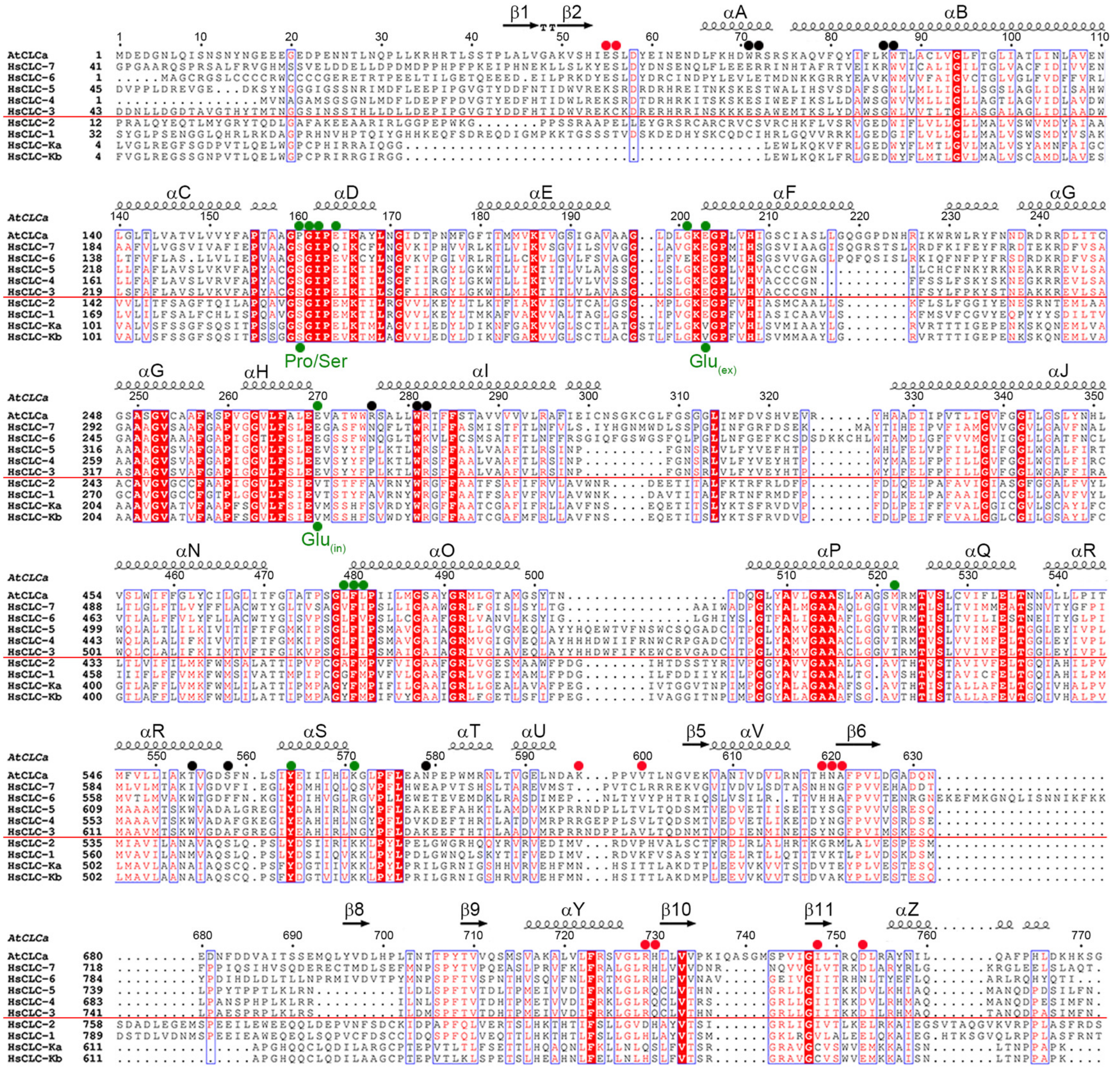

Supplementary Fig. 1 | Sequence alignment of CLC homologues.

At: *Arabidopsis thaliana*; Hs, *Homo sapiens*. The green, red and black dots indicate the residues critical for substrate ion, ATP and PI(4,5)P_2-binding sites in AtCLCa, respectively. Proteins above the red line are CLC transporters, while below the red line are CLC channels.

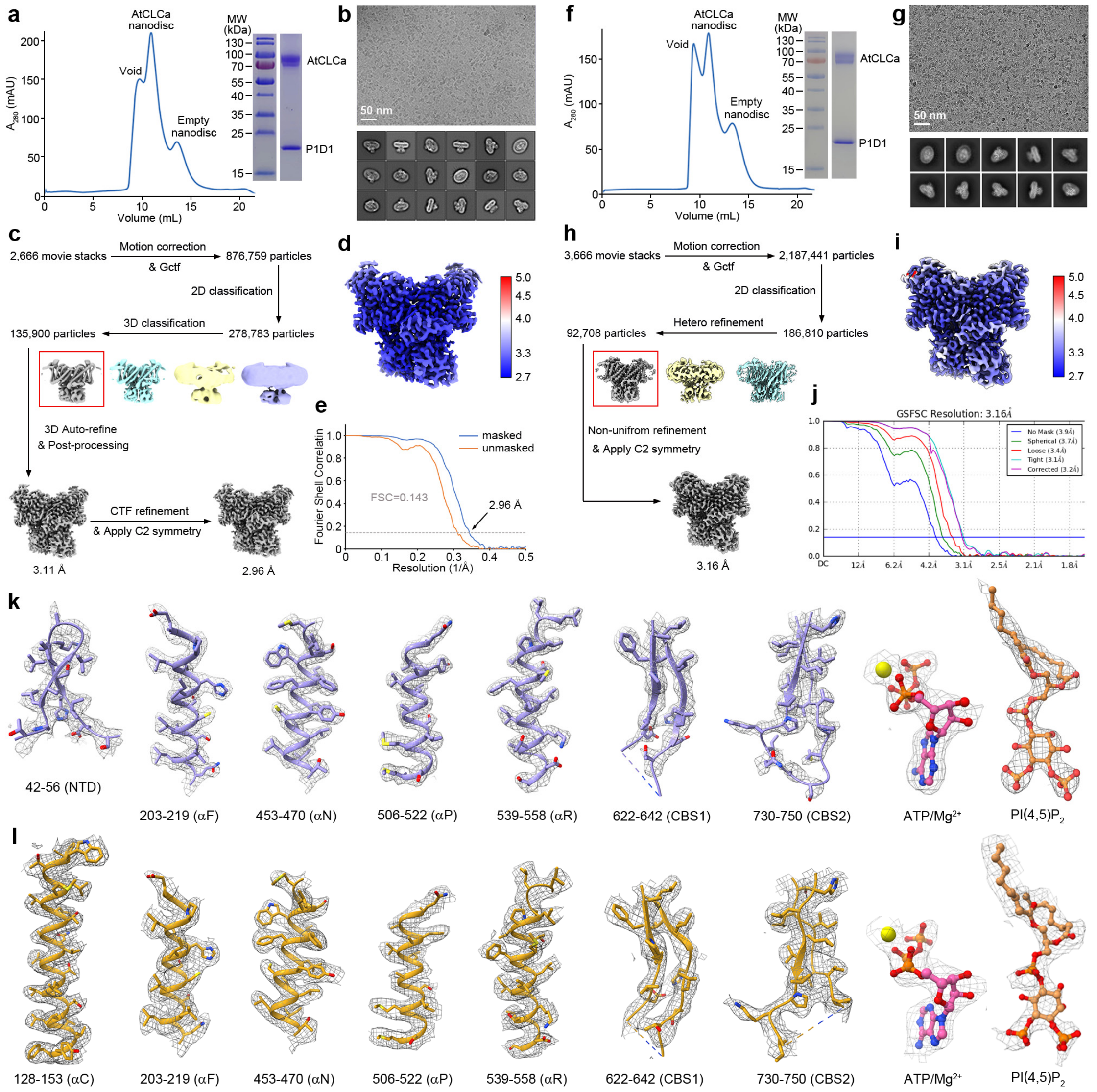

Supplementary Fig. 2 | Cryo-EM Analysis of AtCLCa-Cl$^-$ and AtCLCa-NO$_3^-$.

a, **f** Representative size-exclusion chromatogram from a Superose 200 increase gel filtration and Coomassie-blue-stained SDS-PAGE of the AtCLCa-MSP1D1 nanodiscs. The only anion in the solution buffer used in the purification is Cl$^-$ (**a**) or NO$_3^-$ (**f**). Independent experiments have been repeated for more than three times with similar results. **b**, **g** The representative cryo-EM micrograph and 2D classifications of AtCLCa-Cl$^-$ (**b**) and AtCLCa-NO$_3^-$ (**g**). **c**, **h** Flowchart for data processing of AtCLCa-Cl$^-$ (**c**) and AtCLCa-NO$_3^-$ (**h**). Please refer to Methods for details. **d**, **i** Resolution map for AtCLCa-Cl$^-$ (**d**) and AtCLCa-NO$_3^-$ (**i**). The labels on the right are in unit Å. **e**, **j** The gold-standard Fourier shell correlation (FSC) curves for the 3D reconstructions of AtCLCa-Cl$^-$ (**e**) and AtCLCa-NO$_3^-$ (**j**). **k** EM density of representative segments (gray mesh) in AtCLCa-Cl$^-$. The EM density of residues 42-56 (NTD) is at a contour level of 1 σ, and the other

EM densities are at contour levels of 2 σ. **l** EM density of representative segments (gray mesh) in AtCLCa-NO_3^-. The EM density of PI(4,5)P_2 is at a contour level of 3 σ, and the other EM densities are at contour levels of 4.5 σ. Source data are provided as a Source Data file.

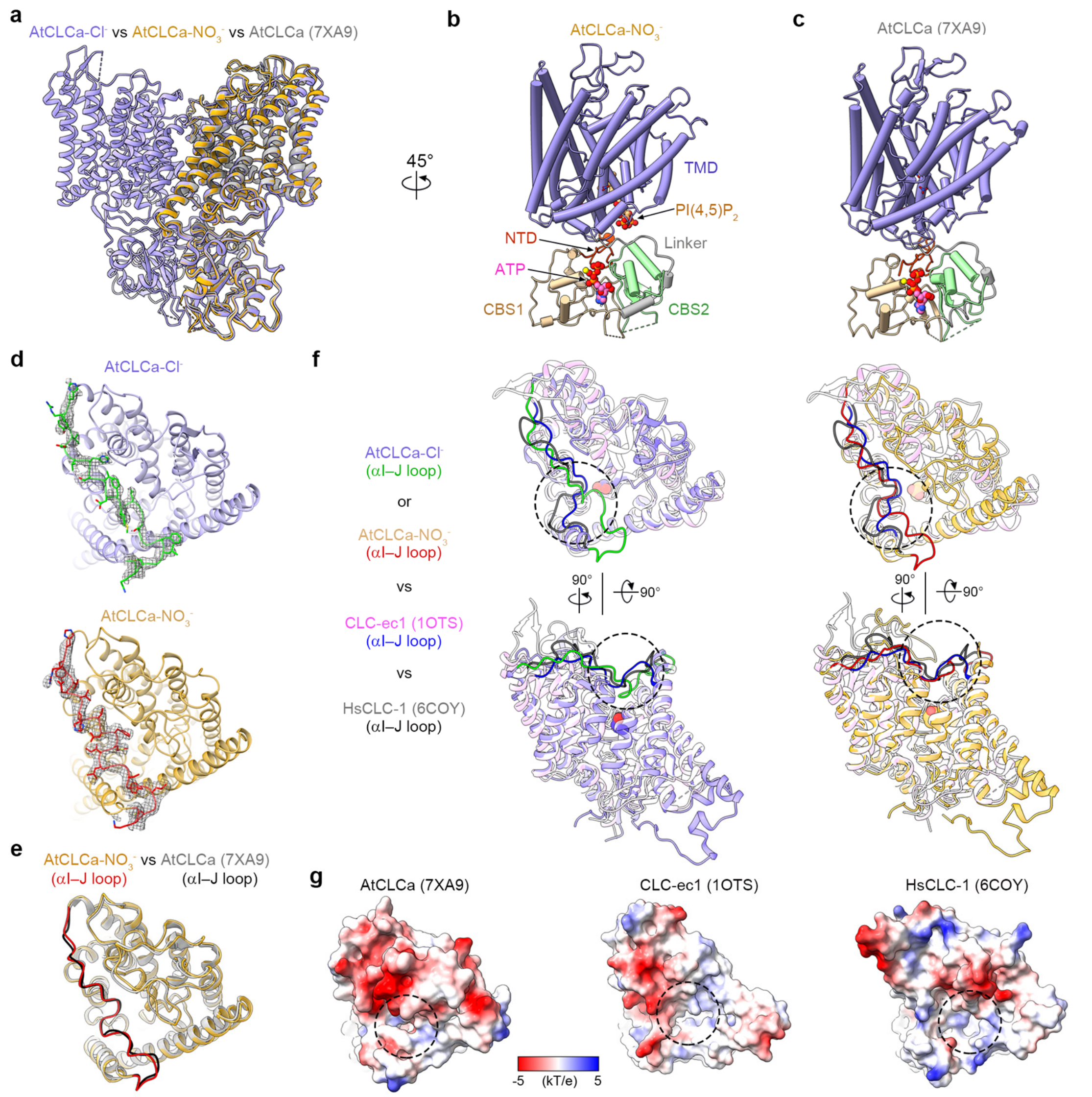

Supplementary Fig. 3 | Conformational difference of CLCs.

a Comparison of AtCLCa-Cl⁻ (blue) and AtCLCa-NO_3^- (golden) and AtCLCa (7XA9, gray) structures. The AtCLCa-NO_3^- protomer and AtCLCa (7XA9) protomer were superimposed onto one protomer of AtCLCa-Cl⁻ dimer. **b** An AtCLCa-NO_3^- protomer colored by domains. NTD, orange red; TMD, blue; Linker, gray; CBS1, beige; CBS2, green; ATP, pink spheres; PI(4,5)P2, beige spheres. **c** An AtCLCa (7XA9) protomer colored by domains as in **b**. **d** EM densities (grey mesh) of the αI–J loop in AtCLCa-Cl⁻ and AtCLCa-NO_3^- structures, which are at contour levels of 1.5 σ and 4.5 σ, respectively. **e** Comparison of the αI–J loop between AtCLCa-NO_3^- and AtCLCa (7XA9). **f** Comparison of the αI–J loop between AtCLCa-Cl⁻ or AtCLCa- NO_3^- with CLC-ec1 (1OTS) and HsCLC-1 (6COY). The CBS domains are hidden for clearance. The side chain of gating glutamate Glu203 of AtCLCa is shown as spheres. The dashed

circle indicates segments of αI–J loop near the outlet of ion transport pathway in luminal side or extracellular side. **g** Electrostatic potential surface of the AtCLCa (7XA9), CLC-ec1 (1OTS) and HsCLC-1 (6COY) protomer. The dashed circle indicates the outlet of ion transport pathway in extracellular side.

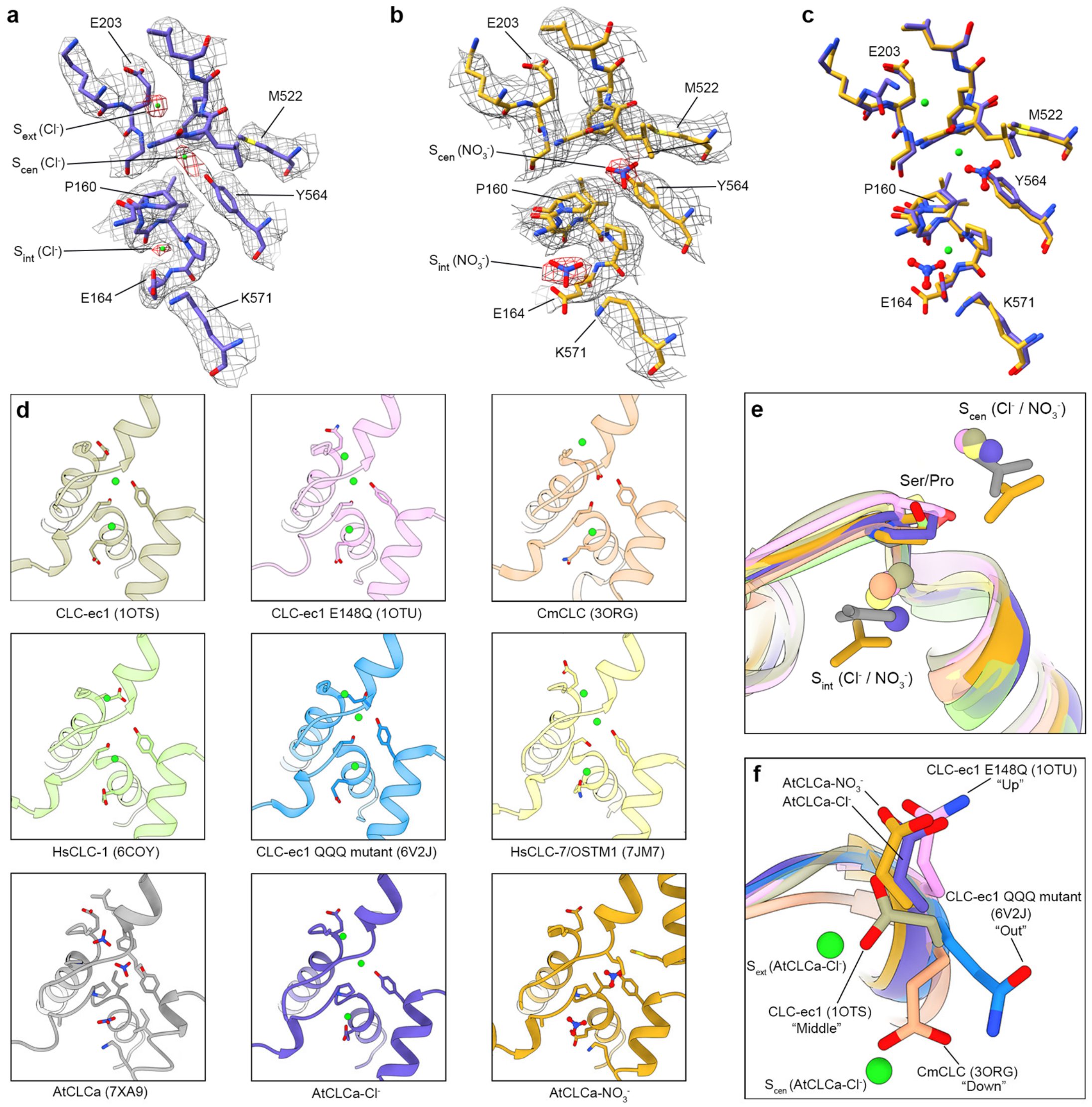

Supplementary Fig. 4 | Substrate binding site.

a, **b** EM densities of substrate-binding site in AtCLCa-Cl^- (**a**) and AtCLCa-NO_3^- (**b**), which are shown at the contour levels of 1.5 σ and 3 σ, respectively. EM densities of Cl^- (**a**) and NO_3^- (**b**) are colored red. **c** Comparison of substrate-binding site in AtCLCa-Cl^- and AtCLCa-NO_3^-. **d** Structures of substrate-binding site of CLCs. Cl^-, green spheres; NO_3^-, balls and sticks. **e** Comparison of the positions of the residue critical to selectivity, proline in AtCLCa and serine in other CLCs, colored as in **d**. **f** Comparison of the gating glutamate conformation between AtCLCa and other representative CLCs, colored as in (**d**).

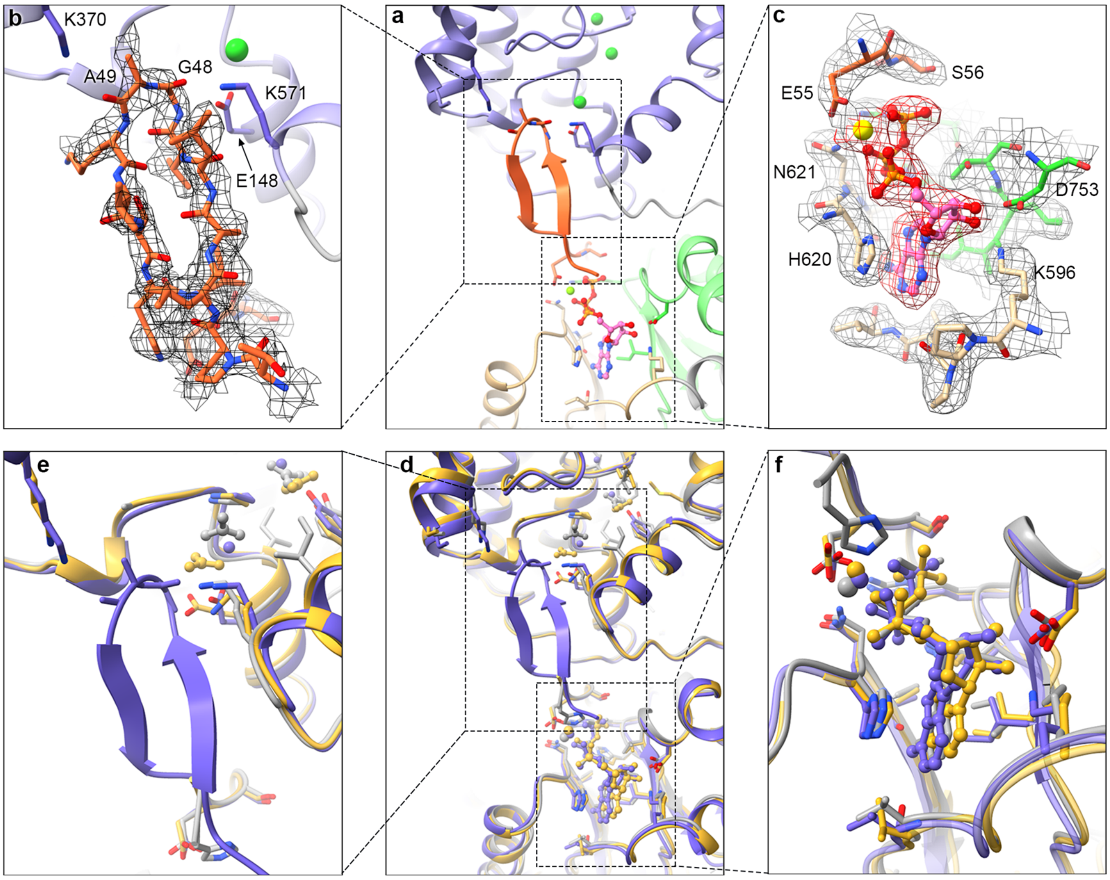

Supplementary Fig. 5 | ATP-binding site.

a N-terminal β-hairpin and ATP-binding site in AtCLCa-Cl^- structure. NTD, orange; TMD, blue; linker, gray; CBS1, beige; CBS2, green; ATP, pink balls and sticks; Cl^-, green sphere; Mg^{2+}, yellow sphere. **b** EM density of N-terminal β-hairpin in AtCLCa-Cl^- structure is show at a contour level of 1 σ. **c** EM density of ATP-binding site in AtCLCa-Cl^- structure is shown at contour level of 2 σ. **d** Comparison of NTD and ATP-binding site between AtCLCa-Cl^- (blue) and AtCLCa-NO_3^- (golden) and AtCLCa (7XA9, gray) structures. **e** A zoom-in view of the comparison of the cytoplasmic entrance to the anion transport pathway. **f** A zoom-in view of the comparison of ATP-binding site.

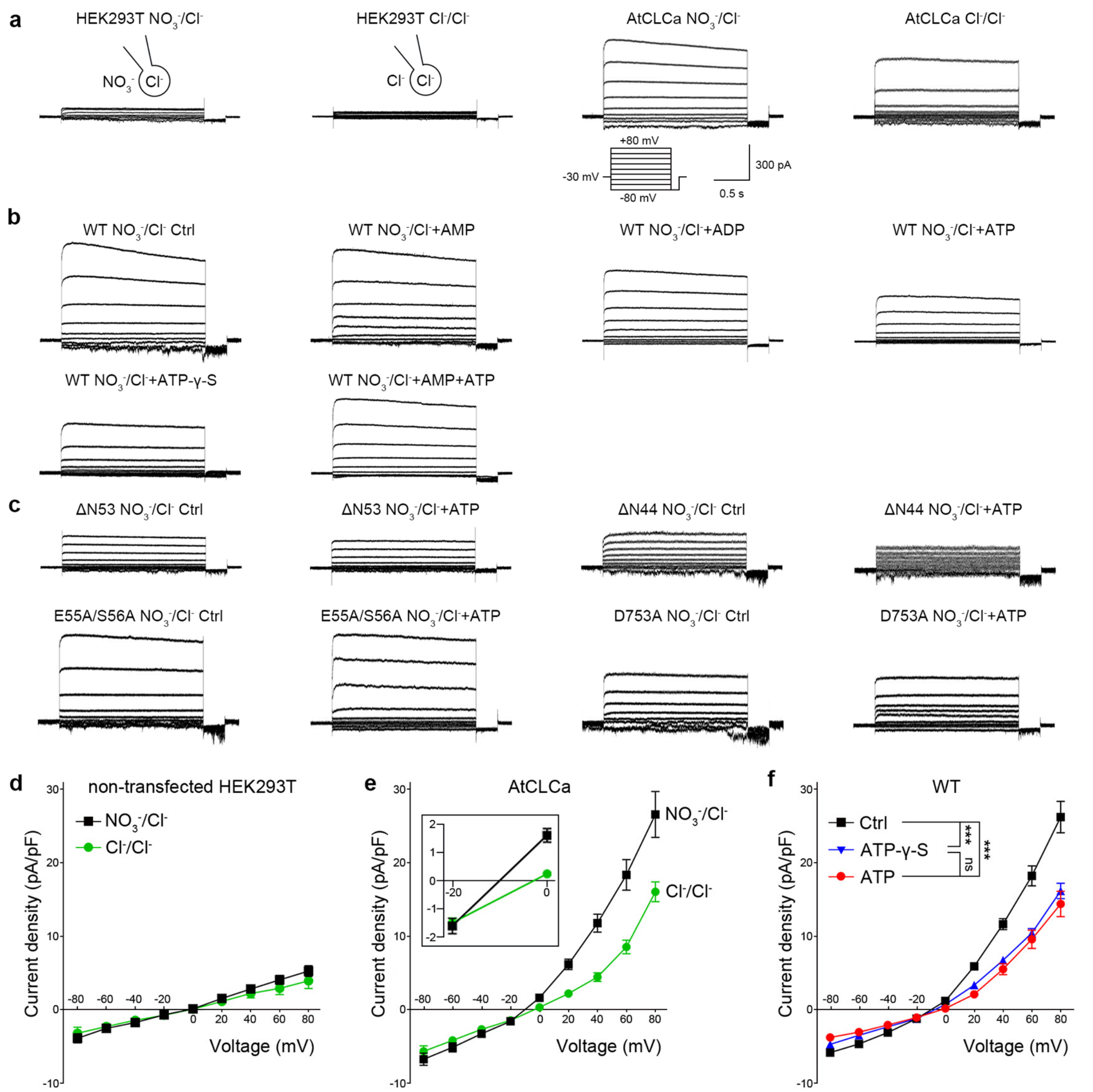

Supplementary Fig. 6 | Electrophysiological analysis of AtCLCa.

a A representative current traces of HEK293T cells untransfected or transfected with AtCLCa in Cl^- and NO_3^- bath solutions. The whole-cell currents were evoked by clamping the HEK293T cells for 2-s voltage pulses from −80 to +80 mV in 20-mV steps followed by a repolarizing step to -80 mV. **b** Representative current traces of wild-type AtCLCa with different adenine nucleotides at a concentration of 5 mM added to the pipette solution and the nitrate bath solution used. Ctrl, control. **c** Representative current traces of AtCLCa ΔN53 mutant, ΔN44 mutant, E55A/S56A mutant and D753A mutant with 5 mM ATP added to the pipette solution and the nitrate bath solution used. Ctrl, control. **d** Comparisons of the *I–V* curves of HEK293T cells without AtCLCa transfection in Cl^- and NO_3^- bath solutions. n = 10 cells for both Cl^- and NO_3^- bath solutions. Data are mean ± s.e.m. **e** Comparisons of the *I–V* curves of HEK293T cells

transfected with AtCLCa in Cl^- and NO_3^- bath solutions. n = 18 cells for both Cl^- and NO_3^- bath solutions. Data are mean ± s.e.m. *Inset*, higher magnification of the *I–V* curves to show reversal potentials. **f** Comparisons of the *I–V* curves of wild-type AtCLCa with different adenine nucleotides at a concentration of 5 mM added to the pipette solution and the nitrate bath solution used. Ctrl, control. n = 20 (Ctrl, nucleotide free), n = 22 (ATP), n = 23 (ATP-γ-S). $P < 0.0001$ (ATP vs Ctrl), $P = 0.0002$ (ATP-γ-S vs Ctrl), $P = 0.4161$ (ATP vs ATP-γ-S).Data are mean ± s.e.m. (n > 20). Two-tailed *t*-tests were used for significance analysis of the current density at +80 mV. *** P < 0.001, ns means no significance difference. Source data are provided as a Source Data file.

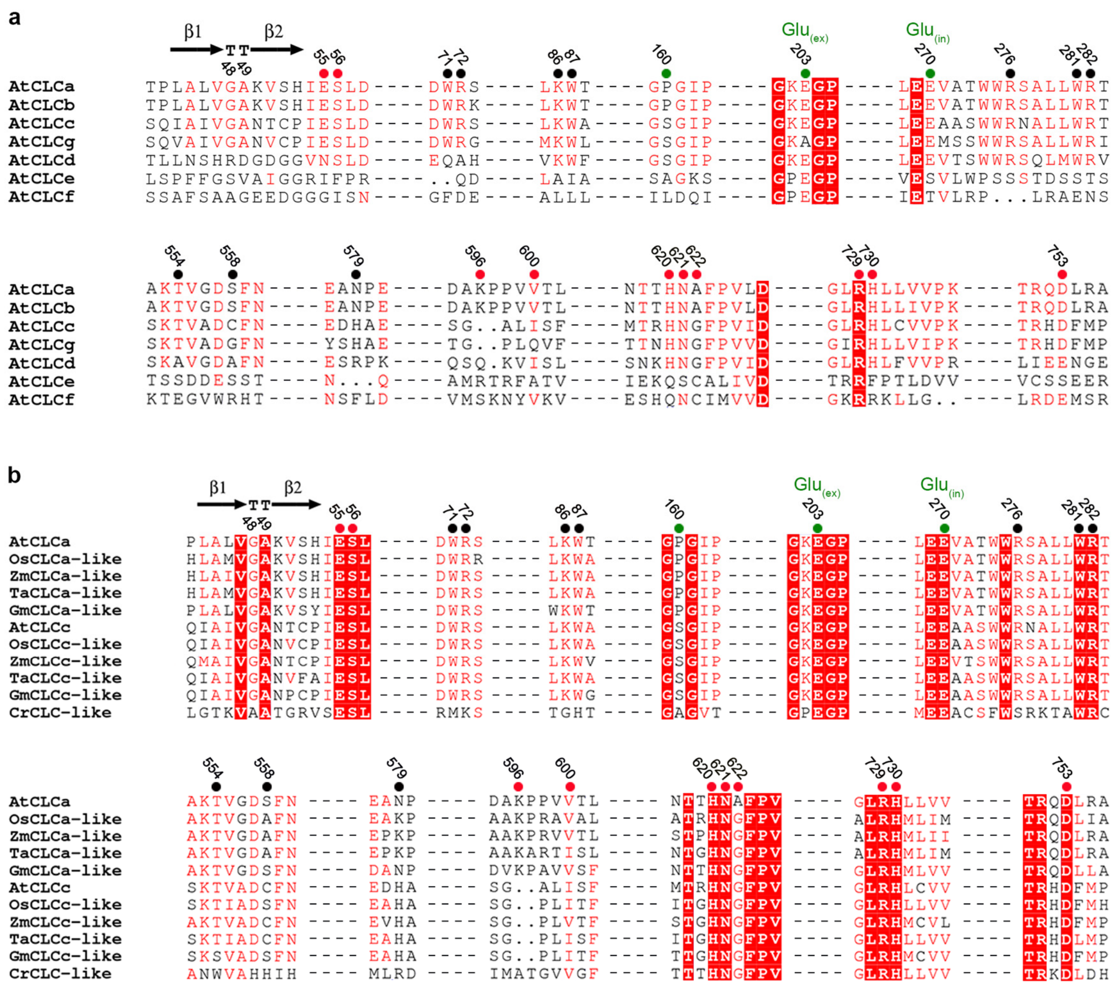

Supplementary Fig. 7 | Sequence alignment of plant CLCs homologues.

a Sequence alignment of *Arabidopsis thaliana* CLC homologues. **b** Sequence alignment of plant CLC homologues. At: *Arabidopsis thaliana*; Os, *Oryza sativa*; Zm: *Zea mays*; Gm: *Glycine max*; Ta, *Triticum aestivum*; Cr, *Chlamydomonas reinhardtii*. The green, red and black dots indicate the residues critical for substrate ion, ATP and $PI(4,5)P_2$-binding sites, respectively.

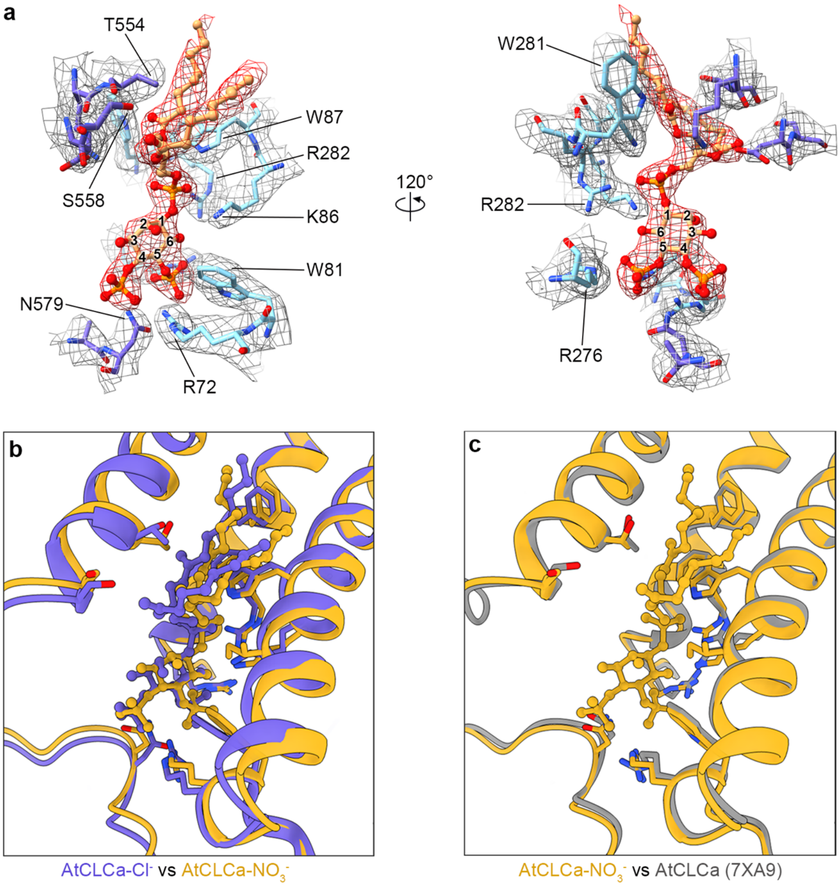

Supplementary Fig. 8 | PIP$_2$-binding site.

a EM density of PI(4,5)P$_2$-binding site in AtCLCa-Cl$^-$ is shown at contour level of 2 σ. EM density of PI(4,5)P$_2$ are colored red. Residues from different protomers are shown as blue and cyan sticks, respectively. **b** Comparison of the PI(4,5)P$_2$-binding site between AtCLCa-Cl$^-$ (blue) and AtCLCa-NO_3^- (golden) structures. **c** Comparison of our PI(4,5)P$_2$-bound AtCLCa-NO_3^- structure (golden) with the recently reported PIP$_2$-free AtCLCa structure (7XA9, gray).

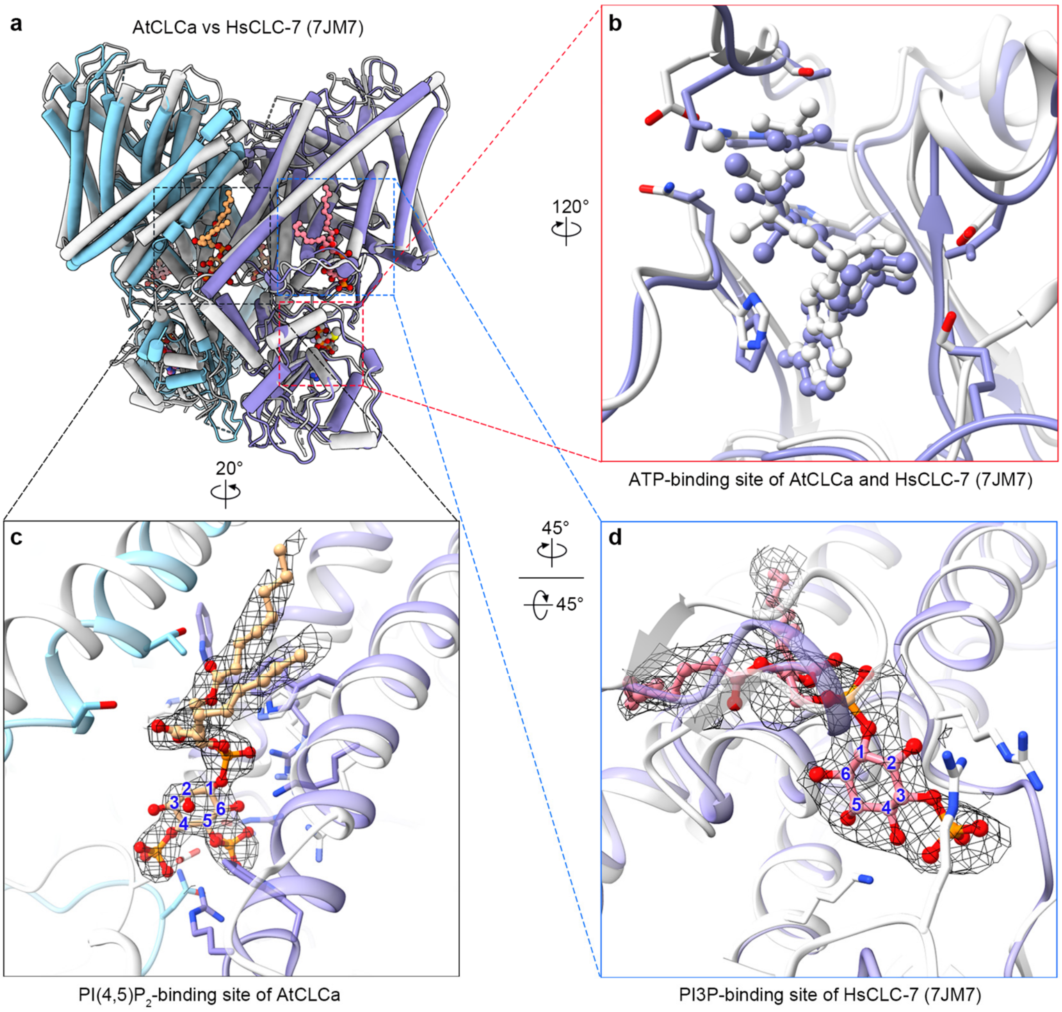

Supplementary Fig. 9 | Comparison of AtCLCa and HsCLC-7 sturctures.

a Comparison of the AtCLCa (cyan and blue) and HsCLC-7 (7JM7, gray) structures. **b** A zoom-in view of the ATP-binding site of AtCLCa and HsCLC-7. **c** A zoom-in view of the PI(4,5)P2-binding site of AtCLCa. EM density (gray mesh) of PI(4,5)P2 in AtCLCa-Cl^- is shown at contour level of 2 σ. **d** A zoom-in view of the PI3P-binding site of HsCLC-7 (7JM7, gray). EM density (gray mesh) of PI3P in HsCLC-7 is shown at contour level of 2 σ.

Supplementary Table 1 |Cryo-EM data collection, refinement and validation statistics

	AtCLCa-Cl^- (EMDB-35299) (PDB 8IAB)	AtCLCa-NO_3^- (EMDB-35300) (PDB 8IAD)
**Data collection and processing**		
Magnification	105,000	105,000
Voltage (kV)	300	300
Electron exposure (e–/Å^2)	56.3	52
Defocus range (μm)	−1.2 to −2.2	−1.2 to −2.2
Pixel size (Å)	0.83	0.832
Symmetry imposed	C2	C2
Initial particle images (no.)	876,759	2,178,441
Final particle images (no.)	135,900	92,708
Map resolution (Å)	2.96	3.16
FSC threshold	0.143	0.143
**Refinement**		
Initial model used (PDB code)	AlphaFold II model	8IAB
Map sharpening *B* factor (Å^2)	-103.7	-98.9
Model composition		
Non-hydrogen atoms	11094	11136
Protein residues	1408	1410
Ligands	12	10
B factors (Å^2)		
Protein	46.48	93.77
Ligand	39.66	97.89
R.m.s. deviations		
Bond lengths (Å)	0.004	0.009
Bond angles (°)	0.789	0.997
Validation		
MolProbity score	1.26	1.72
Clashscore	3.35	5.36
Poor rotamers (%)	0.00	0.25
Ramachandran plot		
Favored (%)	97.28	93.48
Allowed (%)	2.72	6.38
Disallowed (%)	0.00	0.14
